# Mitochondria-associated endoplasmic reticulum membrane (MAM): a dark horse for diabetic cardiomyopathy treatment

**DOI:** 10.1038/s41420-024-01918-3

**Published:** 2024-03-20

**Authors:** Yong Liu, Jin-Ling Huo, Kaidi Ren, Shaokang Pan, Hengdao Liu, Yifeng Zheng, Jingfang Chen, Yingjin Qiao, Yang Yang, Qi Feng

**Affiliations:** 1 Research Institute of Nephrology, Zhengzhou University, the First Affiliated Hospital of Zhengzhou University, 450052 Zhengzhou, P. R. China; 2 Traditional Chinese Medicine Integrated Department of Nephrology, the First Affiliated Hospital of Zhengzhou University, 450052 Zhengzhou, P. R. China; 3 Henan Province Research Center for Kidney Disease, 450052 Zhengzhou, P. R. China; 4 Key Laboratory of Precision Diagnosis and Treatment for Chronic Kidney Disease in Henan Province, 450052 Zhengzhou, P. R. China; 5 Department of Pharmacy, the First Affiliated Hospital of Zhengzhou University, 450052 Zhengzhou, P. R. China; 6 Department of Cardiology, the First Affiliated Hospital of Zhengzhou University, 450052 Zhengzhou, P. R. China; 7 Institute for Biomedical Sciences, Shinshu University, 8304 Minamiminowa, Kamiina, Nagano, 399-4598 Japan; 8 Blood Purification Center, the First Affiliated Hospital of Zhengzhou University, 450052 Zhengzhou, P. R. China; 9 Clinical Systems Biology Research Laboratories, Translational Medicine Center, the First Affiliated Hospital of Zhengzhou University, 450052 Zhengzhou, P. R. China

**Keywords:** Organelles, Cardiovascular diseases

## Abstract

Diabetic cardiomyopathy (DCM), an important complication of diabetes mellitus (DM), is one of the most serious chronic heart diseases and has become a major cause of heart failure worldwide. At present, the pathogenesis of DCM is unclear, and there is still a lack of effective therapeutics. Previous studies have shown that the homeostasis of mitochondria and the endoplasmic reticulum (ER) play a core role in maintaining cardiovascular function, and structural and functional abnormalities in these organelles seriously impact the occurrence and development of various cardiovascular diseases, including DCM. The interplay between mitochondria and the ER is mediated by the mitochondria-associated ER membrane (MAM), which participates in regulating energy metabolism, calcium homeostasis, mitochondrial dynamics, autophagy, ER stress, inflammation, and other cellular processes. Recent studies have proven that MAM is closely related to the initiation and progression of DCM. In this study, we aim to summarize the recent research progress on MAM, elaborate on the key role of MAM in DCM, and discuss the potential of MAM as an important therapeutic target for DCM, thereby providing a theoretical reference for basic and clinical studies of DCM treatment.

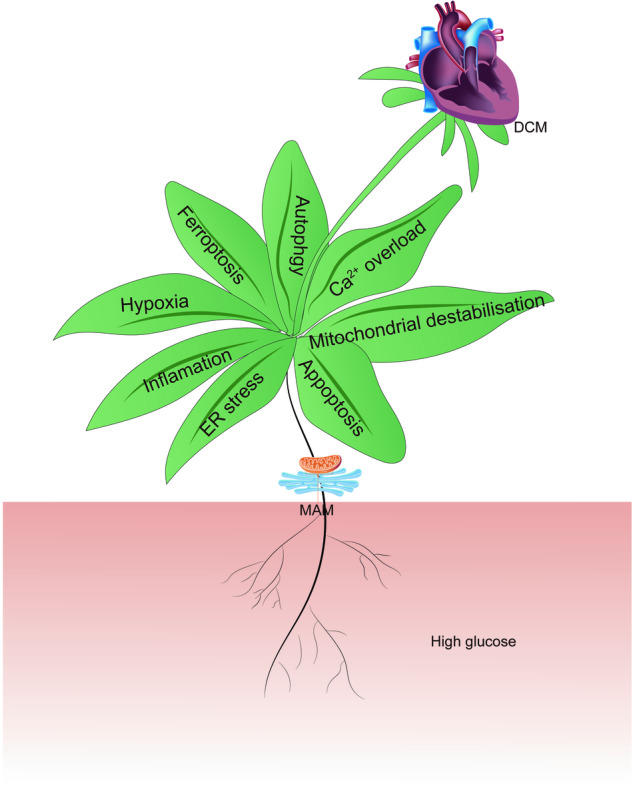

## Facts

- Dysfunction of organelles such as mitochondria and the ER is closely related to the occurrence and development of various heart diseases.
- MAM plays an important role in the pathogenesis of DCM.
- Targeting MAM-related proteins provides a promising approach for preventing the progression of DCM.
- Future studies on the role of MAM in the process of DCM are warranted and highly necessary.

## Open questions

- What are the structural and functional characteristics of MAM?
- What is the role of MAM in the progress of DCM?
- How does the key proteins and complex regulatory mechanisms affect MAM?
- Whether MAM-targeted drugs can be a way to treat DCM?

## Introduction

Diabetic cardiomyopathy (DCM), a progressive heart disease that occurs early in patients with diabetes mellitus (DM), is characterized by cardiomyopathy without the presence of coronary artery disease, hypertension, or valvular heart disease in diabetes and significantly increases the risk of heart failure (HF) [1]. The primary pathological changes of DCM include cardiomyocyte hypertrophy and hyperplasia, deposition of extracellular matrix, thickening of microvascular basement membrane, and interstitial fibrosis [2]. Currently, treatment options for DCM primarily focus on optimizing blood glucose and blood lipid levels and inhibiting oxidative stress [3]. Therefore, exploring the pathogenesis of DCM and identifying effective therapeutic targets are crucial for enhancing the prevention and treatment of this disease.

Approximately 40% of cardiomyocytes are occupied by mitochondria, which provide more than 90% of the ATP needed for normal cardiac function [4]. As a result, the function of the heart is strongly influenced by the condition of the mitochondria, which are often damaged in DCM [5]. Additionally, the contraction and relaxation of heart muscle are regulated by the release of Ca^2+^ within cardiomyocytes, and the sarcoplasmic reticulum (SR) plays a crucial role in this process [6]. The SR is a smooth endoplasmic reticulum (ER) found in cardiac and skeletal muscle fibers [7]. Remarkably, a portion of the Ca^2+^ released from the SR is taken up by mitochondria through close contact between the ER and mitochondria, which stimulates ATP production [8]. The structural connection between the mitochondria and the ER is known as the mitochondria-associated ER membrane (MAM) [9]. Disruption of the MAM structure is a significant factor in the development of HF [10]. Single-cell transcriptional profiling demonstrates MAM-related proteins preferentially accumulate in cardiomyocytes during the initial stages of cardiac hypertrophy but gradually decrease as the disease progresses [11]. In addition to calcium transport, MAM can influence various aspects of the pathological processes involved in DCM, including ER stress, mitochondrial fusion and fission, autophagy, inflammation, oxidative stress, and apoptosis.

In this review, we present an overview of the structure and key resident proteins linked to MAM. Additionally, we provide a summary of the physiological functions of these genes and their roles in the development of DCM.

## Overview of MAM

**Fig. 1:**
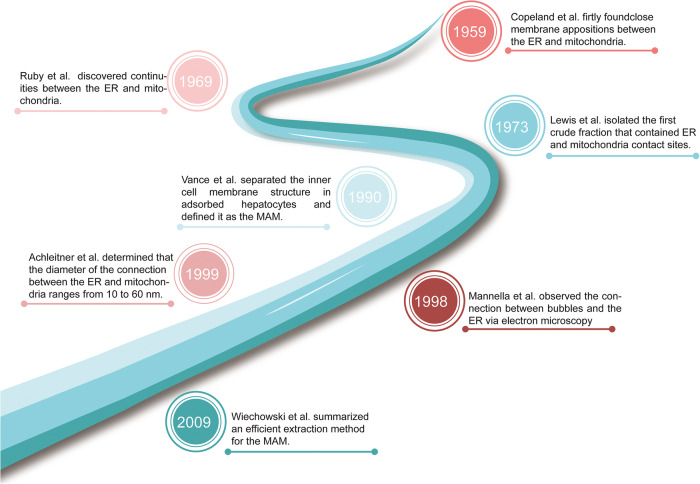
The development timeline of key findings about MAM. In 1959, a close connection between mitochondria and the ER was initially discovered. Ten years later, the continuity between the ER and mitochondria was confirmed through electron microscopy. Moving forward to 1973, the first isolation of an ER–mitochondria contact site was achieved using a crude fraction. In 1990, MAM was officially defined. Subsequently, in 1998, the connection between bubbles and the ER was observed using electron microscopy. In 1999, the diameter range of the connection between the ER and mitochondria was determined. Finally, in 2009, the effective methods for MAM extraction were summarized.

MAM structures refer to physical contacts between the mitochondrial outer membrane and the ER membrane that allow direct communication and exchange of lipids, Ca^2+^, and other molecules between the mitochondria and the ER (Fig. 1). Researchers initially observed a close association between the mitochondria and the ER and speculated that these two organelles might be interconnected. Subsequently, advanced imaging techniques such as electron microscopy were utilized to track and visualize their dynamic movements within cells. To understand the functional significance of the MAM, researchers conducted physiological experiments by manipulation of specific proteins or genes involved in the interaction between mitochondria and the ER. Through these investigations, researchers intensively revealed the existence of a dynamic and functional connection between the mitochondria and the ER, with implications for various cellular processes and the pathogenesis of diseases.

MAMs are dynamic membrane coupling regions that overlap strongly between the outer mitochondrial membrane (OMM) and the ER membrane [12], and their different structures reflect functional diversity [13]. The width of the gap between the ER and OMM varies from 10 to 100 nm [14], and the distance between the ER and mitochondria in MAM differs depending on their functional state. For instance, the MAM involved in Ca^2+^ exchange has a distance between 10 and 25 nm, which is suitable for accommodating Ca^2+^ channels [15]. When ER tubules function in mitochondrial fission, they wrap around mitochondria at a distance of approximately 30 nm [16]. It has been observed that smooth ER membranes can tightly associate with mitochondria, forming contacts that are less than 10 nm in width, thereby facilitating lipid exchange [17]. Therefore, the number, length, and width of the contact zone are important parameters for the involvement of MAM in cellular processes [18].

The composition of MAM is regulated by proteins with various cell biological properties and functions. Resident proteins in the MAM are classified based on their specific functions (Table 1). For example, Ca^2+^ transport-related proteins include inositol voltage-dependent anion channel (VDAC), the molecular chaperone glucose-regulated protein 75 (GRP75) and inositol 1,4,5-trisphosphate receptor (IP3R) [19]; lipid synthesis and transfer-associated proteins, such as protein tyrosine phosphatase interacting protein 51 (PTPIP51) [20]; mitochondrial dynamic regulatory proteins, including dynamin-related protein 1 (DRP1) [21] and mitofusin 2 (MFN2) [22]; and proteins related to insulin signaling, such as GRP75 [23]. The presence of multifunctional proteomes in MAM signifies their crucial roles in regulating cellular homeostasis and biological processes.

**Table 1:** The functional roles of MAM-resident proteins.

Functional types	MAM-related proteins	Abbreviation	Biological functions
Lipid metabolism	Fatty acid CoA ligase 4	FACL4	Immobilization of fatty acids on CoA [125]
	Acy1-Coenzyme A-cholesterol acyltransferase	ACAT	Synthesis of cholesteryl esters [126]
	Phosphatidylserine synthase 1 and 2	PSS1/2	Synthesis of phosphatidyl serine and phosphatidylcholine [127]
	Caveolin-1	CAV1	Regulation of cholesterol efflux [45]
Ca^2+^ hemostasis	Inositol1,4,5-trisphosphate receptor	IP3Rs	Calcium channels in ER [19]
	Voltage-dependent anion channel 1	VDAC1	Calcium uptake channels in mitochondria [19]
	Glucose-regulated protein 75	GRP75	Formation of VDAC1/GRP75/IP3R1 channel complex [19]
	Cyclophilin D	CYPD	Regulates the MAM spatial structure [128]
	Protein tyrosine phosphatase interacting protein 51	PTPIP51	Regulates Ca^2+^ homeostasis [129]
	VAMP Associated Protein B And C	VAPB	Regulates Ca^2+^ homeostasis [129]
	ER resident protein 44	ERp44	Inhibits IP3R [130]
	ER oxireductin1α	Ero1α	Maintains ER redox homeostasis [130]
	Calnexin	CNX	Regulates Ca^2+^ transfer in MAM [6]
	Sarco/ER Ca^2+^ ATPase	SERCA2b	Involves in Ca^2+^ transport into ER [6]
	FUN14 domain-containing protein 1	FUNDC1	Increases mitochondrial Ca^2+^ content [66]
	Glycogen synthase kinase 3β	GSK3β	Regulates organelle Ca^2+^ exchange [31]
	Mitofusin-2	MFN2	Regulates mitochondrial fusion [131]
Mitochondrial dynamics	Dynamin-related protein 1	Drp1	Regulates mitochondrial fission [16]
	Mitofusin-2	MFN2	Regulates mitochondrial fusion [132]
	Inverted formin-2	INF2	Driving initial mitochondrial constriction [16]
	FUN14 domain-containing protein 1	FUNDC1	Regulates mitochondrial fusion and fission [133]
	Mitochondrial calcium uniporter	MCU	MCU knockout decreases mitochondrial division [134]
Inflammation	NOD like receptor (NLR) protein 3	NLRP3	Formation of the NLRP3 inflammasomes and MAMs [135]
	Apoptosis-associated speck-like protein containing a CARD	ASC	Connection of NLRP3 and initiates inflammatory signals [135]
Autophagy	Unc-51-like kinase 1	ULK1	Activation of autophagy downstream pathway [136]
	Beclin 1	BECN1	Enhances the formation of MAMs and autophagosomes [136]
	Autophagy-related 5	ATG5	Autophagosome marker [137]
	Autophagy-related 14	ATG14	Preautophagosome marker [137]
	PTEN induced putative kinase 1	PINK1	Formation of MAMs and autophagosome [138]
Oxidative stress	ER oxireductin1α	Ero1-α	Increases ROS production [139]
	66-kDa isoform of the growth factor adaptor Shc	P66Shc	Induces ROS production [140, 141]
	Glucose-regulated protein 75	GRP75	Reduces mitochondrial ROS [60]
Apoptosis	Mitofusin-2	MFN2	Inhibits cardiomyocyte apoptosis in DCM [63]
	RNA-dependent protein kinase (PKR)-like ER kinase	PERK	Silencing PERK to protect cardiomyocytes [85]
Hypoxia	FUN14 domain-containing protein 1	FUNDC1	Hypoxia induces mitochondrial autophagy [142]
	Glucose-regulated protein 75	GRP75	Hypoxia induces cardiomyocyte apoptosis [94]
Ferroptosis	FUN14 domain-containing protein 2	FUNDC2	FUNDC2 knockout inhibits ferroptosis [114]
	Mitofusin-2	MFN2	MFN2 overexpression inhibits ferroptosis [113]
Copper metabolism	Mitofusin-2	• MFN2	• MFN2 overexpression ameliorates Cu-induced MAM dysfunction [143]

## Basic functions of MAM

**Fig. 2:**
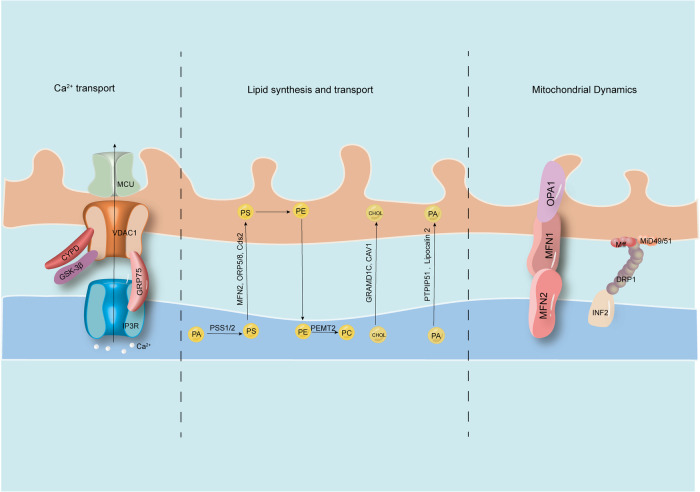
Basic functions of MAM. MAM has been found to be involved in various vital functions, such as Ca^2+^ transport, lipid synthesis and transport, and mitochondrial dynamics. The IP3R-GRP75-VDAC1-MCU axis is a significant channel for calcium transport from the ER to mitochondria. Several proteins, such as PSS1/2, MFN2, ORP2/8, Cds2, GRAMD1C, CAV1, PTPIP51 and lipocalin 2, play critical roles in regulating lipid metabolism. Additionally, proteins such as MFN1/2, OPA1, INF2, DRP1, Mff, and Mid49/51 are essential for regulating mitochondrial fission and fusion.

MAM primarily serve as a hub for Ca^2+^ transport, lipid synthesis and transport, and mitochondrial dynamics (Fig. 2). These crucial functions of MAM have significant implications for the treatment of various diseases.

## Ca^2+^ transport

Excitation-contraction coupling in cardiomyocytes is a complex process that involves changes in the cytoplasmic Ca^2+^ concentration [24]. When the myocardium is excited, extracellular Ca^2+^ enters the cytoplasm through L-type calcium channels on the sarcolemma and transverse tubule [25]. This influx of Ca^2+^ triggers the release of a large amount of stored Ca^2+^ from the SR terminal pool, resulting in a significant increase in the intracytoplasmic Ca^2+^ concentration and ultimately causing contraction of cardiomyocytes. There are two types of calcium-releasing channels, ryanodine receptors (RyRs) and IP3Rs, present in the SR and MAM [26]. These channels, primarily located in the SR, form a tetrameric channel that controls the release of Ca^2+^ from the SR. When activated by Ca^2+^, RyRs allow the influx of extracellular Ca^2+^ to open the channel, leading to the release of a large amount of Ca^2+^ from the SR into the cytoplasm, resulting in myocardial contraction. Notably, contractile activity in cardiomyocytes, which is mediated by Ca^2+^, critically relies on a constant energy supply and sufficient Ca^2+^ buffering, both of which are provided by mitochondria [27]. During the process of contraction, there is a significant increase in mitochondrial Ca^2+^ levels in cardiomyocytes. In this process, IP3R3 interacts with VDAC1, which is located in the OMM, and this interaction is facilitated by the molecular chaperone GRP75. This interaction promotes the uptake of Ca^2+^ by the OMM [28]. In contrast to the high permeability of the OMM, the inner mitochondrial membrane (IMM) primarily transports Ca^2+^ to the mitochondrial matrix through the mitochondrial calcium uniporter (MCU) [29]. MICU1 is considered the most representative Ca^2+^ uptake regulatory protein, and its function is closely related to muscle fiber contraction [30]. Furthermore, several protein chaperones play a role in coordinating calcium transport. For example, glycogen synthase kinase 3β (GSK3β) interacts with IP3Rs to regulate mitochondrial calcium homeostasis in cardiomyocytes [31], and cyclophilin D (CypD) interacts with calcium dynamics in cardiomyocytes and is essential for maintaining proper cardiac function [32].

## Lipid synthesis and transport

The MAM is crucial for various lipid metabolic pathways and is necessary for communication between the ER and mitochondria. Lipids, especially phospholipids (PLs), have important mitochondrial functions. While their synthesis primarily occurs in the ER, mitochondria can synthesize phosphatidylethanolamine (PE), phosphatidylglycerol (PG), and cardiolipin (CL) [33]. Moreover, mitochondria acquire other phospholipids, such as phosphatidylserine (PS), from the ER through MAM, which are key precursors for the synthesis of PE [34]. PS is synthesized by phosphatidylserine synthase (PSS) in the ER and is transported to the cell membrane and mitochondria through lipid transfer proteins at different membrane contact sites [35]. PS decarboxylase (PSD) in the IMM converts PS to PE [36]. In fact, the transfer of PS to mitochondria is a rate-limiting step in PE synthesis. Research has identified proteins, including oxysterol-binding protein-related proteins 5 and 8 (ORP5/8), MFN2, and CDP-diacylglycerol synthase-2 (CDS2), located in MAM that mediate the nonvesicular transport of PS from the ER to mitochondria [37–39]. Knocking out these proteins may lead to excessive cellular lipid accumulation. Furthermore, as a precursor for the synthesis of PG and CL, phosphatidic acid (PA) is synthesized in the ER and transported to mitochondria by MAM. PTPIP51 and lipocalin 2, which are located in MAM, play key roles in regulating the transport of PAs, thereby impacting the synthesis of CLs [20, 40]. Notably, CLs are phospholipids that are specific to mitochondria and crucial for maintaining normal respiratory function. CL deficiency leads to increased vulnerability to lipotoxic hypertrophic cardiomyopathy [41]. Moreover, cholesterol metabolism in the IMM plays an important physiological role, although the cholesterol content in mitochondria is limited. Excessive cholesterol accumulation in mitochondria disrupts mitochondrial activity and impairs the balance of redox reactions within the organelle [42]. This is exemplified by the phenomenon of cholesterol overload leading to the accumulation of oxidized cholesterol molecules known as oxysterols during the early reperfusion phase of myocardial injury [43]. Recent studies have shown that GRAM domain containing 1C (GRAMD1C) may regulate cholesterol transport between the cell membrane and the ER as well as between the ER and mitochondria, inhibit autophagosome synthesis, and downregulate mitochondrial bioenergetics [44]. Knocking out GRAMD1C increases mitochondrial cholesterol content and aerobic respiration [44]. Additionally, caveolin-1 (CAV1), located in the MAM, is a key regulator of cholesterol transport and membrane organization [45]. CAV1 deficiency leads to cholesterol-dependent mitochondrial dysfunction and susceptibility to apoptosis [46]. Long-term consumption of a high-fat and high-sucrose diet (HFHSD) can enhance the affinity between CAV-1 and lipid droplets, promote myocardial lipid accumulation and lipotoxicity, disrupt MAM and mitochondrial morphology, and ultimately result in myocardial cell apoptosis and HF [45].

## Mitochondrial dynamics

The highly dynamic structure of mitochondria allows them to change shape, form, and quantity through fission and fusion, which is crucial for maintaining the normal physiological functions of mitochondria and cells. There is extensive contact between the ER and mitochondria, especially the ER, which induces mitochondrial fission by enveloping a part of the mitochondria. Research by Lewis et al. revealed that spatially stable mtDNA synthesis in the mitochondrial nucleoid of mammalian cells is associated with a small subset of ER-mitochondria contact sites, which coordinate the permission and division of mtDNA replication, distributing newly replicated nucleoids to daughter mitochondria [47]. The main factors mediating fission are Drp1, mitochondrial fission protein 1 (Fis1), and mitochondrial fission factor (Mff) [48]. Drp1 is mainly located in the cytoplasm and is specifically recruited to sites where the ER contacts mitochondria through its receptor Mff, Fis1, and mitochondrial dynamics proteins 49 and 51 (mid49/51), where it forms a helical oligomer that induces membrane constriction and scission [49, 50]. Previously, inverted formin 2 (INF2), located in the ER, was shown to induce actin polymerization and promote the recruitment of DRP1 to mitochondria at ER-mitochondria contact sites [21]. Notably, although Drp1 deficiency prevents mitochondrial fission, the ER-mitochondria contact sites are not disrupted, indicating that Drp1 may not directly tether these two organelles. Mitochondrial fusion is also indispensable in cardiac muscle cells, and this process requires the coordination of outer membrane fusion and inner membrane fusion, with MFN1/2 and optic atrophy 1 protein (OPA1) being relevant molecules located in the OMM and IMM, respectively [51]. MFF1 has higher GTPase activity than MFN2 [52] and can interact with OPA1 [53], while MFN2 is sometimes located on the ER/SR and participates in the connection between the ER/SR and mitochondria through its physical interaction with MFN1 or MFN2 on the outer mitochondrial membrane [22]. Moreover, mitochondrial fusion is regulated by endoplasmic reticulum-associated degradation (ERAD), a protein quality control mechanism that targets proteins in the ER for degradation. Previous studies have demonstrated that loss of ERAD leads to a shorter distance between mitochondria and the ER, increased expression of Sigma1R in MAMs, and enhanced interaction between MFN2 and other MAM proteins. This interaction promotes MFN2 oligomerization, leading to excessive mitochondrial fusion through an unknown mechanism [9].

## The role of MAM in DCM

**Fig. 3:**
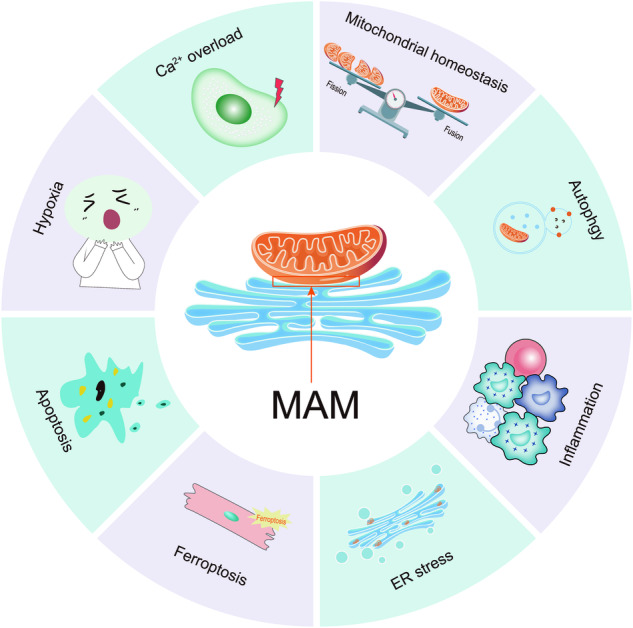
The pivotal roles of MAM in mediating multiple cellular processes in DCM. **The** MAM is a special membrane contact site between the ER and mitochondria, and MAM-resident proteins play key roles in regulating various cellular processes associated with the development of DCM, including Ca^2+^ overload, mitochondrial homeostasis, inflammation, ER stress, hypoxia, apoptosis, and ferroptosis.

Given the significant influence of MAM on various cellular processes, it is crucial to consider their contribution to the development of DCM. In this review, we aim to provide an overview of the pivotal roles of MAM in regulating Ca^2+^ overload, mitochondrial homeostasis, inflammation, ER stress, hypoxia, apoptosis, and ferroptosis in the context of DCM (Fig. 3). Furthermore, we identified the specific MAM-related proteins involved in these processes (Table 2).

**Table 2:** MAM-related proteins involved in DCM.

Functions in DCM	MAM-related proteins
Ca^2+^ overload	CLIC4 [55], MMP-2 [56], AMPD [58], Parkin [59], GRP75 [60], Sig-1R [61], STX17 [144],
Mitochondrial homeostasis	MFN2 [63], Stat3 [64], Drp1, MFN1 [69]
Inflammation	NLRP3 [72], STING [76], CAV1 [80]
ER stress	PERK [85], MFN2 [86]
Hypoxia	PACS2 [96], CaR [95], HIF-1α [94]
Apoptosis	Bcl2 [99], AKAP1 [101], PERK, MFN2 [86]
Ferroptosis	FUNDC1 [115]

## Ca^2+^ overload

The impairment of cardiac function in DCM patients is closely linked to abnormalities in the regulation of Ca^2+^ levels [54]. One interesting observation is the rapid release of Ca^2+^ from the SR following RyR2 activation, which is accompanied by a faster accumulation of Ca^2+^ in the mitochondria [55]. This process may involve certain proteins associated with MAM. Chloride intracellular channel protein 4 (CLIC4), a chloride channel present in the MAM of cardiomyocytes, may increase the rate of Ca^2+^ influx into the mitochondrial domain of the ER under normal physiological conditions [55]. Another enzyme widely expressed in myocardial tissues, matrix metalloproteinase-2 (MMP-2), is predominantly localized to the MAM in cardiomyocytes, where it may modulate Ca^2+^ homeostasis through the control of calreticulin levels [56]. Intriguingly, the activity of MMP-2 decreases significantly in DCM, leading to structural damage and impaired function of the myocardial tissue [57]. Unfortunately, there is currently no direct study on how MMP-2 affects the course of DCM through MAM. Disruption of Ca^2+^ homeostasis in the mitochondria results in Ca^2+^ overload, which impairs mitochondrial function. For example, the upregulation of myocardial adenosine monophosphate deaminase (AMPD) promotes MAM formation, triggering mitochondrial Ca^2+^ overload and cardiac dysfunction in type 2 diabetes [58]. However, this study revealed only a linear relationship between AMPD expression and mitochondrial Ca^2+^ overload in MAM, without providing insight into the specific mechanism by which AMPD regulates Ca^2+^ overload. Several recent studies have focused on targeting Ca^2+^ channels in MAM as a potential approach to prevent Ca^2+^ overload. For example, Wu et al. reported that an inadequate level of Parkin exacerbates high-fat diet (HFD)-induced cardiac remodeling and systolic dysfunction through VDAC1-mediated mitochondrial Ca^2+^ overload [59]. Yuan et al. reported that silencing or knocking out the key gene GRP75 prevented Ca^2+^ overload, alleviated mitochondrial oxidative stress, and protected against atrial remodeling in DCM [60]. Reduced Sigma-1 receptor (Sig-1R) levels in cardiac cells promote mitochondrial fission, decrease mitochondrial Ca^2+^ influx and ER-mitochondrial proximity, and exacerbate cardiomyocyte injury induced by ET-1 [61]. GSK3β is a novel Ca^2+^ regulator located in the SR/ER that interacts with the IP3R Ca^2+^ channel complex. Inhibition of GSK3β reduces Ca^2+^ overload and attenuates myocardial apoptosis induced by ischemia/reperfusion [31]. Moreover, syntaxin 17 (STX17), a scaffolding protein localized on MAM, can facilitate MAM formation by interacting with MCUb, subsequently leading to mitochondrial Ca^2+^ overload, mitochondrial O^2-^ accumulation, and lipid peroxidation. These studies collectively highlight the significance of MAM dysfunction in Ca^2+^ overload. Therefore, targeting the MAM to modulate the transfer of Ca^2+^ between the ER and mitochondria represents a promising approach for mitigating mitochondrial Ca^2+^ overload and attenuating the progression of DCM. However, it remains unclear whether the modulation of cellular Ca^2+^ homeostasis by MAM influences myocardial systolic and diastolic functions in individuals with DCM, and further investigation is needed.

## Mitochondrial destabilization

Mitochondria constantly adjust their shape through fusion and fission in response to changes in energy demand and supply. Studies have shown that, compared to those in control cardiomyocytes, diabetic cardiomyocytes have a decreased mitochondrial size and increased spatial density, which enhances the energy supply of mitochondrial oxidative phosphorylation [62]. However, excessive mitochondrial fission is harmful. Hu et al. reported excessive mitochondrial fission and significantly decreased expression of MFN2 in the hearts of diabetic mice. Reconstruction of MFN2 effectively alleviated DCM by promoting mitochondrial fusion and improving mitochondrial function [63]. In another study, high glucose concentrations decreased the expression of OPA1 and increased its glycosylation. Signal transducer and activator of transcription 3 (STAT3) promotes OPA1 transcription by binding to its promoter region, promoting mitochondrial fusion and preventing DCM [64]. Notably, STAT3 was found to be located in MAM rather than in mitochondria [65]. Furthermore, the cardiac-specific loss of Fundc1, a protein involved in MAM formation, eliminated diabetes-induced MAM formation, preventing mitochondrial calcium overload, mitochondrial fragmentation, and cell apoptosis while improving mitochondrial functional capacity and cardiac function [66]. Transient receptor potential vanilloid 1 (TRPV1), a nonselective cation channel activated selectively by capsaicin (CAP), alleviates endothelial dysfunction and DCM in diabetic rats [67]. TRPV1 can promote MAM formation and attenuate myocardial hypertrophy injury by influencing the adenosine monophosphate-activated protein kinase (AMPK)-MFN2 pathway, which exerts beneficial effects on mitochondrial homeostasis [68]. Lon protease 1 (LonP1), a highly conserved mitochondrial matrix protease, significantly reduces MAM formation when it is ablated, leading to mitochondrial fragmentation and dilated cardiomyopathy-related heart failure. This may be because LonP1 ablation in cardiomyocytes promotes OPA1 processing and Drp1 expression and reduces MFN1 expression to enhance mitochondrial fission [69]. In addition, MAM affects mitochondrial homeostasis through Ca^2+^ transmission. Wu et al. conducted a study revealing that FUNDC1 plays a crucial role in the formation of MAM in the heart through IP3Rs. Disruption of the interaction between FUNDC1 and IP3R leads to suppressed Fis1 expression and mitochondrial fission by reducing the binding of the cAMP response element-binding protein to the Fis1 promoter [28]. Subsequent studies also revealed that the excessive mitochondrial fission caused by the increased expression of FUDC1 in diabetic hearts can be suppressed by activating AMPK [66]. The above studies highlight the critical role of MAM in mitochondrial homeostasis.

## Inflammation

Chronic myocardial inflammation is one of the main manifestations of DCM [70]. Nucleotide-binding oligomerization domain-like receptor family pyrin domain-containing 3 (NLRP3) senses signals of microbial infection and cellular damage and further forms multiprotein complexes called “inflammasomes” to induce inflammatory responses [71]. Numerous studies have demonstrated the involvement of NLRP3 inflammasome activation in the pathogenesis of DCM. Importantly, targeting NLRP3 inflammasome activation has promising potential for delaying the progression of DCM [72]. Initially, NLRP3 is localized in the ER membrane and cytoplasm of macrophages. Once activated, it translocates to MAM, where it interacts with its adapter apoptosis-associated speck-like protein containing a caspase recruitment domain (ASC), further facilitating its activation by MAM-derived effectors [73]. Interestingly, inhibition of stimulator of interferon genes (STING) has been found to reduce NLRP3 inflammasome activation [74]. STING, an adapter protein involved in innate immunity, primarily resides in the ER and MAM [75]. Ma et al. reported that high fat intake in diabetes leads to an increase in mitochondrial ROS production, mitochondrial damage, and mtDNA leakage in cardiomyocytes. Additionally, it also activated the cytoplasmic DNA sensor cyclic GMP–AMP synthase (cGAS), which promoted the translocation of STING to the Golgi apparatus. As a result, IRF3 and NF-κB were activated, leading to inflammation and apoptosis and ultimately resulted in DCM [76]. Activation of the cGAS-STING axis is influenced by intracellular Ca^2+^ levels [77]. Abnormal mitochondrial dynamic is believed to cause increased Ca^2+^ exchange between the ER and mitochondria, leading to the retention of STING in the microdomain of the MAM. This retention limits the translocation of STING to the Golgi apparatus and subsequently affects its ability to mediate interferon signal transduction [78]. Furthermore, lipid metabolism disorders are also important contributors to inflammation [79]. CAV1, a crucial regulator of lipid metabolism that is highly concentrated in the MAM of cardiomyocytes, possesses anti-inflammatory properties in DCM [80]. Regrettably, this study failed to deeply investigate the mechanisms of CAV1 and MAM in DCM. In summary, MAM relies on key molecules such as STING and NLPR3 to mediate the inflammatory response in DCM. The regulation of Ca^2+^ and lipid homeostasis between the ER and mitochondria, facilitated by MAM, plays a crucial role in triggering inflammatory responses.

## ER stress

ER stress is an early event in DCM and can be triggered by various conditions such as hyperglycemia, insulin resistance, inflammation, accumulation of free fatty acids, and increased ROS production [81]. Initially, ER stress compensates for the impaired ER function through the unfolded protein response, which is primarily regulated by three ER stress sensor proteins: glucose-regulated protein 78 (GRP78), RNA-dependent protein kinase-like ER kinase (PERK), activated transcription factor 6 (ATF6), and inositol-requiring enzyme 1 alpha (IRE1α) [82]. However, prolonged or excessive ER stress leads to metabolic dysfunction and apoptosis. Studies have shown that downregulation of ATF6 and PERK levels can inhibit ER stress-induced cardiomyocyte apoptosis in DCM [83]. During ER stress, IRE1 interacts with Sig-1R to promote dimerization. As a calcium receptor, when ER calcium is depleted, Sig-1R dissociates from GRP78, thereby promoting the transfer of calcium to mitochondria through IP3R [84]. Previous studies have demonstrated that PERK is abundantly present in the ER and MAM in high glucose-cultured cardiomyocytes. The PERK-mediated signaling pathway plays a significant role in the apoptosis induced by ROS-mediated ER stress in DCM [85]. In addition, activation of the PERK pathway under high glucose conditions often leads to the downregulation of MFN2 level and a weakened interaction with MFN2 [12]. Yuan et al. discovered that decreasing MFN2 expression alleviated ER stress in atrial myocytes induced by high glucose, which is primarily due to an increased distance between the ER and mitochondria in MFN2 knockdown atrial myocytes, resulting in a decrease in the transfer of Ca^2+^ from the ER to the mitochondria [86]. According to a recent study, it has been found that an excessive amount of ER stress can result in abnormal transfer of calcium in the mitochondria, which in turn leads to mitochondrial damage. This damage causes an increase in the production of mitochondrial reactive oxygen species, which activates the NLRP3 inflammasome and NLRP3 inflammasome in cardiomyocytes, ultimately resulting in scorched death [87]. These findings highlight the interconnectedness of ER stress, Ca^2+^ transport, and inflammation through MAM crosstalk in DCM.

## Hypoxia

The heart has a high energy demand due to its contractile function and relies primarily on mitochondrial fatty acid oxidation for energy production [88]. Notably, free fatty acids (FFAs), as a source of energy for heart muscle, are less effective because they require approximately 10% more oxygen than glucose to produce an equivalent amount of adenosine triphosphate [89]. However, under hyperglycemic conditions, fatty acid β-oxidation in the heart increases, while glucose oxidation decreases, further exacerbating myocardial hypoxia [90]. In addition, insufficient blood supply is also an important factor leading to cardiac hypoxia. Although DCM itself does not directly cause cardiac ischemia‒reperfusion, the decreased tolerance of diabetic hearts due to abnormal myocardial function may worsen myocardial injury and increase the scope and severity of myocardial infarction when cardiac ischemia‒reperfusion occurs [91]. Hypoxia-inducible factor 1 (HIF-1) is a key regulator of the cellular response to hypoxia [92]. Under conditions of high glucose and hypoxia, HIF-1α and FOXO3a synergistically induce cardiomyocyte death [93]. Moulin et al. found that chronic intermittent hypoxia-activated HIF-1α, disrupted the MAM structure, impaired Ca^2+^ transfer between the ER and mitochondria, and ultimately induced cardiomyocyte apoptosis [94]. Moreover, SR-mitochondrial Ca^2+^ signaling is subject to regulation by the calcium-sensing receptor (CaR) under hypoxic conditions. CaR activation during cardiac cell hypoxia-reoxygenation induces SR Ca^2+^ release and increases Ca^2+^ uptake into the mitochondria through MAM [95]. In addition, hypobaric hypoxia was found to downregulate phosphofurin acidic cluster sorting protein 2 (PACS2), which in turn disrupted the formation of MAM and hindered the transfer of Ca^2+^ between the ER and mitochondria, ultimately resulting in cardiomyocyte injury and heart dysfunction [96]. The above studies confirmed MAM played an important role in the cellular response to hypoxia. However, further study is needed to reveal the specific mechanism of MAM in DCM-related cardiac hypoxia.

## Apoptosis

Myocardial apoptosis contributes to the occurrence and progression of DCM. Tissue biopsy studies have revealed that apoptosis in the hearts of diabetes is 85 times higher than that of nondiabetic hearts, suggesting an increased sensitivity of myocardial cells to cell death in diabetes [97]. Mitochondria and ER are important organelles involved in mediating cell apoptosis, and MAM typically regulates cell apoptosis by modulating intracellular Ca^2+^ concentration, mitochondrial function, ER stress, and inflammation [98]. In diabetic mice, there is a decrease in the expression of brain and muscle arnt-like protein 1 (Bmal1) in the heart, leading to an increased formation of MAM [99]. The reduced expression of Bmal1 inhibits the transcription level of Bcl2 and weakens the interaction between Bcl2 and IP3R, thereby promoting the release of Ca^2+^ from the ER to mitochondria via IP3R. Ultimately, this activates mitochondrial-mediated cell apoptosis and promotes the development of DCM. However, this condition can be alleviated by overexpression of Bmal1 [99]. Notably, Bcl2 is primarily located in the ER and translocates to MAM and mitochondria during cell apoptosis induction [100]. Furthermore, in cardiomyocytes under high-glucose conditions, PERK accumulates in both the ER and MAM, receiving stimulation from ROS released into these structures, thereby inducing ER stress and apoptosis in myocardial cells [71]. As an upstream regulator of PERK, the silencing of MFN2 can prevent mitochondrial Ca^2+^ overload-mediated mitochondrial dysfunction, thereby reducing ER stress-mediated myocardial apoptosis [86].In addition, the lack of A-kinase anchoring protein 1 (AKAP1) in DCM impairs mitochondrial respiratory function and enhances the production of ROS, leading to increased apoptosis of myocardial cells [101]. Interestingly, AKAP1 has also been found to localize to MAM [102]. Moreover, it has been reported that high glucose conditions cause MAM aberrations and mitochondrial dysfunction by upregulating PACS2, IP3R2, FUNDC1, and VDAC1, thereby leading to cardiomyocyte apoptosis [103]. These studies indicate that MAM is involved in the apoptotic process of DCM.

## Autophagy

In the myocardium, maintaining appropriate levels of autophagy is crucial. Excessive autophagy is harmful and will lead to diabetic cardiomyocyte damage and death [104]. Both inhibition and overactivation of autophagy cause structural and functional dysfunction in the diabetic heart [105]. It reported that the destruction of the MAM structure was a significant contributor to abnormal autophagy in hearts [106]. MAM served as a platform for autophagy-related proteins to carry out their biological functions. For example, saturated fatty acids block autophagy by accumulating saturated lysophosphatidic acids in MAM, which aggravates vascular calcification [107]. In addition to lipid metabolism, maintaining myocardial autophagy levels also depends on Ca^2+^ homeostasis [106]. Wei et al. discovered that TRPV1 activated Ca^2+^ influx, phosphorylated AMPK, and promoted cardiomyocyte autophagy [108]. Moreover, MAM is involved in autophagy regulation by maintaining mitochondrial homeostasis. Studies have demonstrated that Drp1 disruption leads to mitochondrial elongation, mitophagy inhibition, and mitochondrial dysfunction, ultimately contributing to cardiac dysfunction [109]. However, STX17 recruits the kinase CDK1 through its SNARE domain to phosphorylate DRP616 at the Ser1 site in MAM, subsequently promoting mitophagy in cardiomyocytes [1]. In summary, MAM plays a role in regulating autophagy through various pathways such as lipid metabolism, Ca^2+^ homeostasis, mitochondrial fission, and fusion. Considering the distinct effects of autophagy in different stages of DCM, it is important to target MAM at the appropriate intervention time.

## Ferroptosis

Ferroptosis, an iron-dependent form of cell death caused by lipid peroxidation, is involved in regulated cell death and controlled by integrated oxidation and antioxidant systems [109]. In the context of DCM, the development of oxidative stress and impairment of antioxidant systems are fundamental mechanisms [110]. Therefore, targeting oxidative stress sources or endogenous antioxidant defense systems, as well as removing ROS, may be effective approaches for treating DCM. Recent studies have found that the expressions of SLC7A11 and glutathione (GSH) are significantly downregulated in the hearts of DCM mice [111]. This downregulation can disrupt normal cell function, enhance lipid peroxidation, and contribute to iron-dependent cell death, which is an important factor in DCM development [111]. It is suggested that MAM may play a role in the mechanisms underlying ferroptosis. For instance, dysfunction of MAM may lead to an imbalance in Ca^2+^ transport between the ER and mitochondria, thereby driving ferroptosis [112]. It has been found that acute exposure to arsenic impairs MAM function, possibly by weakening the interaction between MFN2 and PERK in lung epithelial cells, thereby inducing ferroptosis [113]. In doxorubicin-induced cardiomyopathy, a recent study has discovered that the OMM protein FUNDC2 promotes ferroptosis by regulating the stability of SLC25A11 and mitochondrial GSH levels [114]. Moreover, another OMM protein FUNDC1 demonstrated its role in regulating cell ferroptosis in DCM. A study revealed that a deficiency of FUNDC1 increased sensitivity to heart remodeling and functional impairment caused by short-term HFD exposure. This may be attributed to the regulation of ACSL4-mediated cell ferroptosis [115]. Therefore, these studies indicate that MAMs are instrumental in the occurrence of ferroptosis in DCM and further research is needed to clarify the mechanisms by which MAMs contribute to this process.

## Outlook

DCM is a significant contributor to disability and mortality among diabetic patients [1]. The interaction between organelles is involved in the occurrence and development of various heart diseases. As important subcellular structures, MAM is closely related to the functional status of myocardial cells. The density, length, and thickness of MAM is influenced by cell metabolic status and stress levels [116]. Therefore, MAM may strengthen the connection between the ER and mitochondria and affect the fate of cardiomyocytes by changing the structure and composition of MAM-resident proteins under different pathophysiological conditions. Recent studies have shown that MAM is involved in Ca^2+^ overload, mitochondrial homeostasis, autophagy, inflammation, ER stress, apoptosis, ferroptosis, and other cellular processes in DCM. Therefore, targeting MAM has become a potential method for DCM treatment. For example, ferulic acid protected the integrity of MAM, inhibited apoptosis, and improved cardiomyopathy in diabetic rats [103]. Paeonol promoted Opa1-mediated mitochondrial fusion by activating Stat3, which might be a promising strategy for DCM treatment [117]. Similarly, cordycepin was demonstrated to protect diabetic hearts by upregulating MFN2 expression and promoting mitochondrial fusion, thus safeguarding against myocardial ischemia/reperfusion injury [118].

With a deeper understanding of the role of MAMs, certain previous perceptions are now being controversial. A ketogenic diet (KD) is widely used by diabetic patients; however, Tao et al. found that while a KD improved the metabolic indices of *db/db* mice, it inhibited the proliferation of T-regulatory cells (Tregs), impaired diastolic function and exacerbated ventricular fibrosis [119]. This effect might be mediated primarily by inhibiting MAM and blocking fatty acid metabolism through the inhibition of IL-33/ST2L signaling [119]. Previously, the inhibition of excessive mitochondrial division was considered an effective means for alleviating DCM [63]. Recent studies have revealed that there are two types of mitochondrial divisions: intermediate and peripheral divisions. Intermediate divisions predominantly occur during the active phase of cell growth and division and are closely linked to the ER; peripheral divisions take place in unfavorable cellular environments and are mainly associated with lysosomes [120]. Therefore, the types of mitochondrial division involved in the pathological state of DCM need to be further investigated to determine whether targeting MAM is effective in inhibiting excessive mitochondrial fission in disease states. However, the specificity of targeting MAM also deserves attention. For example, metformin is commonly used as a first-line treatment for diabetes [121]. In addition to its hypoglycemic effect, it can exert cardioprotective effects by activating the AMPK pathway and improving mitochondrial function [122]. Interestingly, AMPK plays a key role in MAM [123]. Therefore, metformin might be a promising drug for the treatment of DCM via MAM. Maya et al. showed that metformin improved blood glucose levels and insulin sensitivity in HFHSD mice; however, it could not prevent the alteration of MAM Ca^2+^ coupling in cardiomyocytes and ameliorate the progression of DCM [124]. These findings further underscore the critical role of MAM Ca^2+^ coupling as a potential therapeutic target for DCM and highlight the essential drug specificity of targeting MAM.

In conclusion, this review systematically summarizes the structure and function of MAM, examines the various cellular processes influenced by MAM, and assesses the potential of MAM as a key therapeutic target for DCM. However, it should be noted that most of the studies on MAM and DCM involve animal models and preclinical experiments. Therefore, further investigations of MAM-related proteins and their potential mechanisms in cardiac diseases are needed to provide new perspectives for the clinical treatment of DCM.

## Data Availability

The data used to support the findings of this study are included within the paper.
